# Learning Health System Implementation: Building a Hub-and-Spoke Model for Hypertension Management Through the QI Hub

**DOI:** 10.1007/s11606-025-10152-1

**Published:** 2026-01-13

**Authors:** Gabriel Alain, Laura J. Rush, Riley Summers, Najhee Purdy, Jennifer Eramo, Sarah Jonaus, Keri Cooper, Shalina Nair, Crystal Smarr, Ashley Sneed, Anne VanBuren, Lauren Teuschler, Jamileh Alain, Catherine Quatman-Yates

**Affiliations:** 1 LIFT Lab, College of Medicine, The Ohio State University, Columbus, OH USA; 2 School of Health and Rehabilitation Sciences, The Ohio State University, Columbus, OH USA; 3 CATALYST, College of Medicine, The Ohio State University, Columbus, OH USA; 4 College of Public Health, The Ohio State University, Columbus, OH USA; 5 College of Medicine, The Ohio State University, Columbus, OH USA; 6 General Internal Medicine Division, The Ohio State University Wexner Medical Center, Columbus, OH USA; 7 Quality and Operations, The Ohio State University Wexner Medical Center, Columbus, OH USA; 8 Department of Family Medicine, The University of Vermont Health Network, Burlington, VT USA; 9 Family and Community Medicine Division, The Ohio State University Wexner Medical Center, Columbus, OH USA; 10 Division of Physical Therapy, School of Health and Rehabilitation Sciences, The Ohio State University, Columbus, OH USA

**Keywords:** learning health system, hub-and-spoke model, hypertension, primary care, quality improvement, implementation science, blood pressure control, healthcare infrastructure, population health management

## Abstract

**Background:**

In July 2022, the Ohio Department of Medicaid (ODM) launched a statewide Regional Quality Improvement (QI) Hub program using a hub-and-spoke model to embed Learning Health System (LHS) capabilities in primary care. The Ohio State University (OSU) Hub piloted the approach across ten affiliated sites, aiming to raise overall hypertension control by ≥ 10 percentage points and promote more consistent levels of hypertension control across patient populations by June 2027. This report describes the first 18 months post-implementation.

**Methods:**

A mixed-methods, formative evaluation followed the Exploration-Preparation-Implementation-Sustainment (EPIS) framework. The hub provided centralized data extraction (bi-weekly Epic pulls), an R Shiny dashboard, and tailored coaching on Plan-Do-Study-Act cycles (PDSA) while participating in statewide learning collaboratives. Blood pressure (BP) control rates were tracked with statistical process control (SPC) charts. Site-level logistic regressions tested interaction effects between demographics (non-Hispanic Black vs. White) and implementation period.

**Results:**

Among 22,563 hypertensive adults (73,264 encounters), the baseline centerline was 72.9% controlled BP. Two SPC shifts, April 2024 to 77.3% and July 2024 to 79.1%, produced a + 6.2 percentage-point (+ 8.5%) relative improvement, equating to ~ 4542 additional controlled BP encounters beyond baseline expectations. Site-level absolute changes ranged from −3.9% to + 14.6%. Across sites, six narrowed and four widened the Black-White BP control gap. Site-level sensitivity analysis showed no significant change in the gap. Qualitative data highlighted the importance of near real-time feedback and flexible coaching; staffing turnover constrained progress in several under-resourced clinics.

**Conclusion:**

Implementing a hub-and-spoke LHS model within a large academic health system was feasible and associated with sustained gains in hypertension control over 18 months. Centralized analytics, adaptive QI support, and statewide peer learning were key enablers, whereas workforce instability remained a barrier. Early results support the model’s potential to build LHS capacity in primary care, with ongoing work needed to strengthen uniform impact and long-term sustainability.

**Supplementary Information:**

The online version contains supplementary material available at https://doi.org/10.1007/s11606-025-10152-1.

## INTRODUCTION

Healthcare systems worldwide face increasing demands to deliver high-quality, cost-effective care while improving patient outcomes, patient experiences, clinician experiences, population health, and health equity.^1–4^ Learning Health System (LHS) approaches offer a promising pathway to meet these expectations. An LHS is “a system in which science, informatics, incentives, and culture are aligned for continuous improvement and innovation, with best practices seamlessly embedded in the delivery process and new knowledge captured as an integral by‑product of care.”^5^ Robust LHS infrastructures integrate electronic health record (EHR) data, analytics, quality improvement (QI) methods, and change‑management strategies so that near real‑time information feeds rapid‑cycle learning, research, and practice improvement.^6–8^ Numerous studies have demonstrated that such infrastructures can accelerate improvements in chronic‑disease outcomes, patient safety, and cost.^7^

In primary care, the LHS framework holds particular relevance because of the potential to address common issues such as polypharmacy management^8,9^ and coordination of care across multiple specialty areas.^8^ By enabling access to near real-time insights, an effective LHS infrastructure can provide primary care physicians and health system leaders with information that can be used to inform and prioritize support for chronic disease management.^10^ Core LHS features include strong inter‑organizational partnerships; a shared vision and governance; systems and processes that enable iterative and sustainable improvement; feedback to frontline patients, clinicians, and health services; and multidisciplinary teams embedded in routine care, all underpinned by EHR or linked data registries.^7^

A hub-and-spoke organizational structure offers one mechanism for operationalizing LHS principles at scale. With a hub-and-spoke model, a central hub provides standardized knowledge assets, analytic support, and QI coaching to multiple spoke practices, enabling network-level learning and local adaptation.^11,12^ Spoke sites can then adapt the support they receive from the hub to their specific contexts and patient populations, enabling a more unified networked approach and localized improvement simultaneously.^13^

The Ohio Department of Medicaid (ODM) recently launched a statewide Regional QI Hub initiative to advance population health and reduce disparities through scalable, data-driven quality improvement embedded within a LHS framework.^14^ The strategy aimed to align measures, data, and resources across clinics, managed care entities, and state agencies by establishing Regional QI Hubs housed within Ohio’s Colleges of Medicine (COM). These hubs were designed to leverage academic infrastructure to promote QI best practices, implement innovative care models, and support widespread adoption of chronic disease management strategies in primary care.

Crane et al. provided a comprehensive account of the rationale, design, implementation strategy, and lessons learned from the first year of Ohio’s statewide Regional QI Hub initiative.^15^ The initiative established six Regional QI Hubs at The Ohio State University (OSU), University of Cincinnati (UC), University of Toledo (UT), Northeast Ohio Medical University (NEOMED), Case Western Reserve University (CWRU), and Ohio University (OU). The Ohio COM Government Resource Center (GRC) served as the coordinating center, overseeing statewide project management, QI training, and data infrastructure. A statewide learning collaborative supported the exchange of data definitions, analytic tools, and implementation strategies across institutions, while each hub developed operational infrastructure tailored to its local partners. The six hubs were divided into trios (Table 1) focusing on hypertension or diabetes.

**Table 1:** **Collaborative Trios Of Ohio COM’s Participating In ODM’s Regional QI Hub. The Table Below Shows The Breakdown of Each Chronic Disease Trio, The Name Of The COM, Its Acronym, And Its Location**

City	College of Medicine	Acronym
**Hypertension trio**		
Cincinnati	University of Cincinnati	UC
Columbus	The Ohio State University	OSU
Toledo	University of Toledo	UT
**Diabetes trio**		
Athens	Ohio University	OU
Cleveland	Case Western Reserve University	CWRU
Rootstown	Northeast Ohio Medical University	NEOMED

This paper reports on early findings from the first 18 months of OSU’s Regional QI Hub, implemented as part of the broader ODM collaborative. It focuses on the launch and implementation of a hub-and-spoke model during Wave 1, which piloted hypertension management across ten affiliated primary care sites.

## METHODS

### Guiding Conceptual and Reporting Frameworks

**Figure 1:**
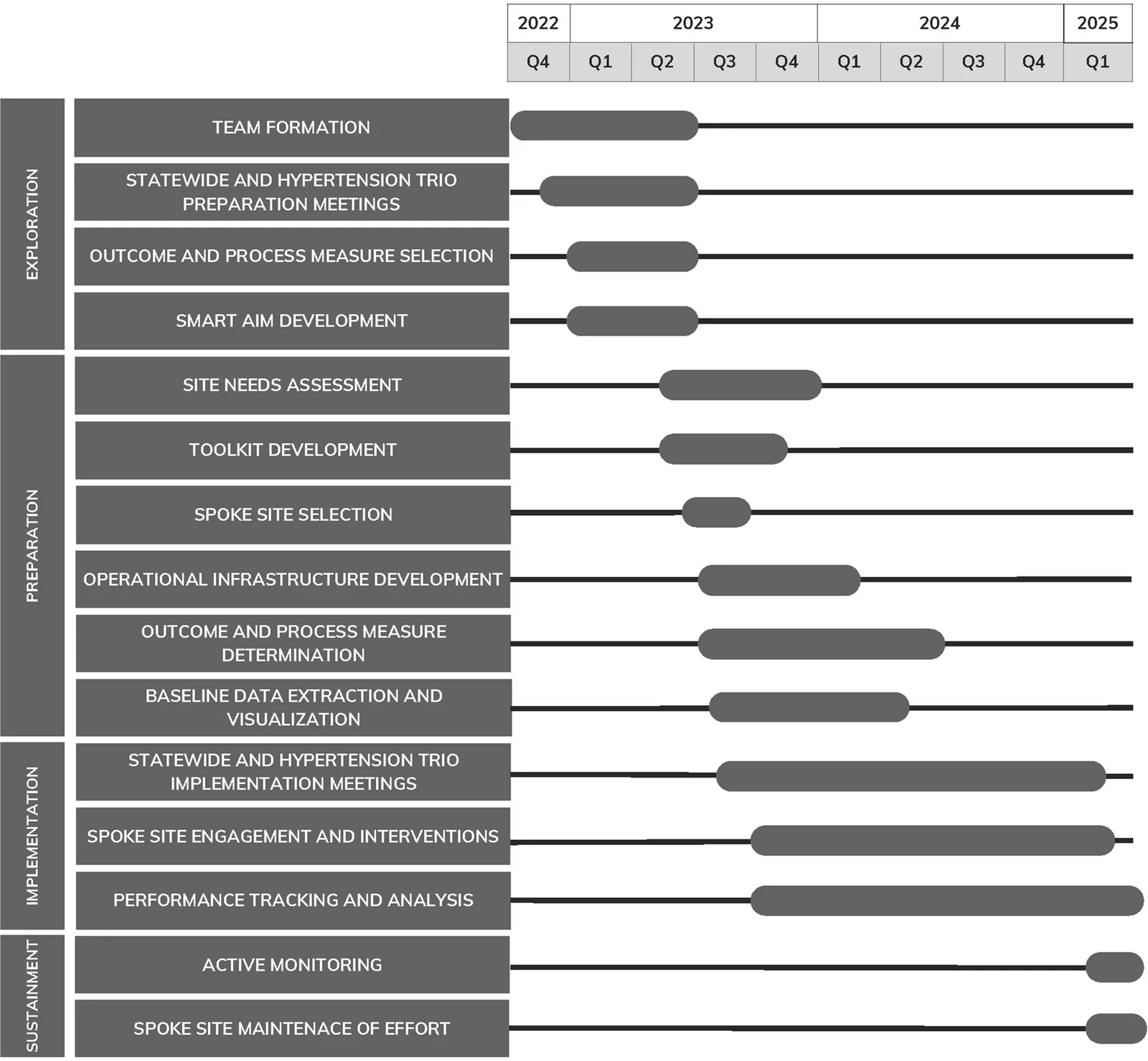
**Gantt chart of implementation timeline and key activities for regional QI Hub-and-spoke model development. This chart shows activities during the exploration, preparation, implementation, and sustainment phases with their corresponding time of occurrence between the third quarter of the 2022 calendar year (2022 Q3) and the second quarter of the 2025 calendar year (2025 Q2).**

This report was developed using a mixed-methods formative evaluation approach, guided by the Exploration, Preparation, Implementation, Sustainment (EPIS) framework.^16^ The EPIS framework was selected for its structured approach to delineating project phases and its capacity to account for the complex, multilevel factors, both inner and outer contextual, that influence implementation processes and outcomes.^16,17^ Our reporting approach was further organized and aligned with the Standards for Quality Improvement Reporting Excellence (SQUIRE 2.0) guidelines.^18^ Figure 1 presents a Gantt chart illustrating the timeline of major project activities across phases.

### Ethical Considerations

This QI project was reviewed by our Office of Responsible Research Practices, which determined it did not meet the federal definition of human subject research and therefore did not require review. Human ethics and consent to participate declarations are not applicable.

### Context and Setting

Our Regional QI Hub was housed within OSU’s COM, which operates under the broader university structure and maintains a distinct operational framework from the OSU Wexner Medical Center (OSUWMC). While administratively situated in the COM, the QI Hub was designed to run in parallel with, and integrate into, OSUWMC’s QI infrastructure. OSUWMC encompasses a wide network of General Internal Medicine and Family Medicine primary care practices distributed across central Ohio. Geographic and organizational diversity offered both opportunities and challenges for implementing standardized QI strategies across heterogeneous practice settings, staffing models, and patient populations.

All ten primary care sites (spokes) participating in our initial Wave 1 were affiliated with OSUWMC and utilized a common EHR system (Epic, Verona, WI). Spoke sites were selected based on a combination of factors, including a high percentage of patients covered by Medicaid, patient volume, pre-existing collaborative relationships, and operational feasibility for piloting data extraction and visualization processes. Our selection strategy aimed to include a diverse mix of high- and low-volume clinics serving Medicaid populations in order to align with funder requirements and create opportunities for cross-site learning.

### Exploration Phase

#### QI Hub Team Formation and Statewide Collaboration

Activities for the exploration phase began in October 2022 with the formalization of our OSU QI Hub team and development of an operating structure capable of supporting spoke sites effectively. The hub team comprised physicians and advanced practice providers from primary care, informaticists, a data scientist, program managers, professional staff, and QI coaches (*n* = 17 total). During this phase, ODM had designated hypertension as the primary condition for initial pilot efforts based on its high prevalence, significant public health impact, and persistent treatment gaps.

The GRC initiated monthly meetings with all Regional Hub teams to foster a statewide learning community for sharing approaches and lessons learned. In December 2022, our team aligned with the UC and UT hubs to form the hypertension control trio subgroup, which began meeting monthly to explore evidence-based interventions, determine shared operational definitions for outcomes, and identify successful care delivery models.

#### SMART Aim Development

Beginning in January 2023, each Regional Hub developed specific, measurable, achievable, relevant, and time-bound (SMART) aims tailored to their local contexts. The OSU QI Hub team established two primary SMART aims: (1) improve the overall percentage of adult patients with a diagnosis of hypertension whose blood pressure is controlled by at least 10 percentage points and (2) improve the percentage of Hispanic and non-Hispanic Black adults whose blood pressure is controlled by at least 14 percentage points by June 2027. The June 2027 timeline aligned with ODM’s staged rollout plan across three waves over approximately 5 years.

### Preparation Phase

#### Needs Assessment

The preparation phase began with dual objectives: building functional QI Hub infrastructure and selecting and preparing ten spoke sites for hypertension improvement efforts. A comprehensive multi-stakeholder needs assessment was conducted through site visits by clinical staff and project managers from the Hub team. These visits incorporated workplace observations (“Gemba walks”) of patient-facing processes from beginning to end, in order to yield qualitative insights into site-specific workflows, potential facilitators, and implementation barriers.

#### QI Hub Infrastructure Development

To address identified needs, we established focused teams for implementation, education, comprehensive patient care, data management, and strategic alignment. Funds were allocated for clinical time buyouts to enable key stakeholder participation as champions. The implementation team developed evidence-based toolkits for improving hypertension control rates. The education team created a centralized website housing QI templates and training videos. The comprehensive patient care team identified existing resources while conducting ongoing analyses of social and community barriers. The data team supported EHR extractions and dashboard development, while the strategic alignment team fostered collaboration between the QI Hub and broader health system QI efforts.

#### Outcome and Process Measure Development

The hypertension trio collaboratively defined standardized operational measures to enable data aggregation from spoke sites to hub and statewide levels. The primary outcome aligned with the NCQA electronic Clinical Quality Measure (eCQM) for blood pressure (BP) control (CMS165v11).^19^ An encounter-based approach was selected to reflect real-time clinical activity and avoid challenges related to patient attribution and registry maintenance. For each reporting period, the denominator included all unique adults aged 18–85 years with documented essential hypertension (ICD-10-CM: I10) and at least one qualifying encounter. The numerator comprised those whose most recent BP at that site was < 140 mmHg systolic and < 90 mmHg diastolic. Only BP readings recorded during in-clinic encounters at participating primary care sites were included; home or specialty-clinic measurements were excluded. Additional process measures included re-check rates, follow-up scheduling, medication management practices, and BP control stratified by demographic characteristics.

#### Baseline Data Infrastructure

Baseline measurement was set from July 1, 2022, to August 31, 2023. Data were extracted bi-weekly from the institution’s Epic-based warehouse and submitted via REDCap to the GRC using a consensus-developed common data model. The GRC populated a centralized R Shiny dashboard that displayed performance metrics at the state, hub, and spoke levels (SupplementalFig. 1). Statistical process control (SPC) methods^20^ were used to establish baseline performance and control limits, distinguishing routine variation from true system-level change.^21^

### Implementation Phase

#### Coordinated Launch and Site Engagement

The implementation phase commenced with coordinated launches across all ten spoke sites, each receiving a $5000 budget to support local implementation activities. Structured kickoff meetings introduced the improvement toolkit and adapted evidence-based interventions to each clinic’s specific context. Core intervention components were adopted from the 2017 American College of Cardiology/American Heart Association (ACC/AHA) hypertension guidelines,^22^ including standardized BP measurement protocols, team-based care models, patient self-management support strategies, and population health management techniques.

#### Flexible QI Support and PDSA Implementation

Following kickoff, the Hub provided ongoing, flexible QI support tailored to each site’s preferences and needs. Engagement frequency varied significantly, with some sites opting for intensive engagement every 2–3 weeks while others preferred check-ins every 4–6 weeks. Support encompassed coaching on Plan-Do-Study-Act (PDSA) cycles, assistance with data review and interpretation, and active facilitation of cross-site learning through sharing of successful strategies and lessons learned.

PDSA cycle selection and focus were primarily site-driven and tailored to local contexts and operational realities. While the Hub team developed an initial menu of evidence-based PDSA focus areas (e.g., improving BP measurement accuracy, enhancing reliability of BP rechecks), sites were encouraged to identify their own improvement priorities. Some practices adapted suggested options while others developed novel PDSAs based on unique needs or existing improvement efforts. The Hub’s role remained consistent, providing data support, QI methodology coaching, and resource connection regardless of PDSA origin.

#### Continued Statewide and Trio Collaboration

Throughout implementation, active participation continued in both statewide learning collaboratives and hypertension trio meetings. The GRC-facilitated structure promoted the spread and scale of successful strategies, enabled innovation sharing, and discussed system-level facilitators and barriers. Monthly statewide meetings included all Regional Hubs, while additional monthly trio meetings focused specifically on hypertension interventions. Learnings and best practices from these collaboratives were integrated and disseminated to individual spoke sites.

#### Data Collection and Analysis

##### Qualitative Data Collection and Integration

Qualitative data regarding implementation context, barriers, facilitators, and PDSA cycles were systematically collected through multiple sources: Gemba walk notes, fishbone diagrams, Hub team meeting records, and PDSA documentation. These data were synthesized to provide contextual understanding of quantitative findings and elucidate implementation processes. The integration of qualitative and quantitative data enabled a comprehensive evaluation of both intervention effectiveness and implementation factors.

##### Statistical Analysis Approach

Our analysis focused on key metrics including centerline percent changes and absolute changes in hypertension control, comparing baseline performance to outcomes observed during the 18-month implementation period, which spanned from October 2023 through April 2025. Control charts were monitored for special cause variation using SPC methodology. To assess whether observed changes in demographic differences were driven by site-level effects rather than systematic intervention consequences, a site-level sensitivity analysis was conducted. For each site, separate logistic regression models were fitted with BP control as the dependent variable, using race, implementation period, and their interaction as independent variables. Statistical analysis was performed using R (v.4.3.1; R Core Team, 2023).^23^

### Sustainment Phase

Sustainment was defined prospectively as a 6-month period during which sites demonstrated stable or improving hypertension control rates, no special cause variation on SPC analysis, and ongoing implementation of at least one core intervention. During this phase, the QI Hub planned to transition to a monitoring and support role, continuing bi-weekly data collection and maintaining dashboard access. Sites could request consultation as needed, and sustainability planning focused on reinforcing local ownership and integrating effective practices into system-level QI infrastructure.

## RESULTS

### Participant Demographics

A total of 22,563 adult patients with hypertension were included across the 10 participating primary care sites. The overall report population had a mean age of 59.8 years and was 53.2% female. A total of 73,264 patient encounters took place over the 18-month period. Additional patient demographics for each participating site are provided in Table 2.

**Table 2:** **Demographics of Patients By Site. The Table Below Shows Race/Ethnicity, Age (In Years), And Type Of Insurer For All Patients At The Time of Their Index Encounter During The 18-month Implementation Period. The Percentage Of Encounters Attributed To Each Demographic Group During The Same Period Is Indicated**

	**Site 1**	**Site 2**	**Site 3**		**Site 4**	**Site 5**	**Site 6**	**Site 7**	**Site 8**		**Site 9**	**Site 10**	**Total**
**Characteristic**				**Number of individuals (% of encounters)**									
**Race/ethnicity**													
Hispanic Black	0 (0.0%)	1 (0.1%)	5 (0.2%)		2 (0.1%)	5 (0.2%)	1 (0.1%)	7 (0.3%)	6 (0.2%)		8 (0.3%)	1 (0.0%)	36 (0.2%)
Hispanic White	5 (1.0%)	18 (0.8%)	22 (1.0%)		31 (1.0%)	18 (0.6%)	4 (0.8%)	13 (0.5%)	14 (0.6%)		14 (0.3%)	13 (1.0%)	152 (0.7%)
Non-Hispanic Black	21 (4.6%)	389 (18.1%)	312 (12.1%)		297 (8.4%)	416 (14.7%)	87 (14.2%)	505 (26.9%)	1077 (32.0%)		2223 (68.3%)	561 (33.8%)	5888 (28.7%)
Non-Hispanic White	364 (85.0%)	1370 (68.5%)	1911 (74.0%)		2536 (76.0%)	2289 (75.7%)	561 (77.3%)	1172 (59.8%)	2259 (60.3%)		879 (24.7%)	939 (56.2%)	14,280 (60.6%)
Other	44 (9.4%)	237 (12.5%)	294 (12.6%)		466 (14.7%)	254 (8.7%)	59 (7.6%)	214 (12.4%)	247 (6.9%)		233 (6.4%)	159 (9.1%)	2207 (9.8%)
**Age (in years)**													
18–34	9 (2.0%)	122 (5.8%)	113 (3.8%)		137 (3.2%)	75 (2.3%)	77 (12.1%)	141 (7.3%)	110 (3.0%)		211 (5.3%)	74 (4.0%)	1069 (4.4%)
35–64	263 (60.0%)	1098 (50.3%)	1346 (49.3%)		1600 (46.7%)	1078 (32.5%)	442 (59.0%)	1349 (70.1%)	1978 (54.2%)		2078 (61.0%)	1002 (57.7%)	12,234 (52.6%)
65 +	162 (38.1%)	795 (43.9%)	1085 (46.9%)		1595 (50.1%)	1829 (65.2%)	193 (29.0%)	421 (22.6%)	1515 (42.8%)		1068 (33.7%)	597 (38.3%)	9260 (43.0%)
**Insurer**													
Medicaid	34 (7.4%)	187 (10.1%)	208 (9.0%)		265 (9.3%)	172 (5.7%)	68 (11.0%)	350 (19.4%)	300 (9.1%)		875 (26.6%)	271 (16.2%)	2730 (13.3%)
Medicare	147 (35.7%)	723 (38.9%)	1022 (44.5%)		1437 (45.6%)	1692 (61.4%)	198 (30.9%)	377 (20.3%)	1436 (41.6%)		1204 (38.6%)	632 (42.5%)	8868 (42.0%)
Private/commercial	237 (52.9%)	1052 (49%)	1260 (44.8%)		1573 (43.4%)	1079 (31.8%)	428 (55.5%)	1102 (56.1%)	1782 (46.8%)		1149 (31.1%)	724 (39.1%)	10,386 (42.3%)
Self/pay	2 (0.3%)	7 (0.4%)	7 (0.2%)		7 (0.2%)	12 (0.4%)	5 (0.5%)	9 (0.5%)	12 (0.3%)		8 (0.2%)	3 (0.3%)	72 (0.3%)
Other	14 (3.6%)	46 (1.6%)	47 (1.5%)		50 (1.6%)	27 (0.7%)	13 (2.0%)	73 (3.7%)	73 (2.2%)		121 (3.5%)	43 (1.9%)	507 (2.1%)

### Trajectory of Hypertension Control

**Figure 2:**
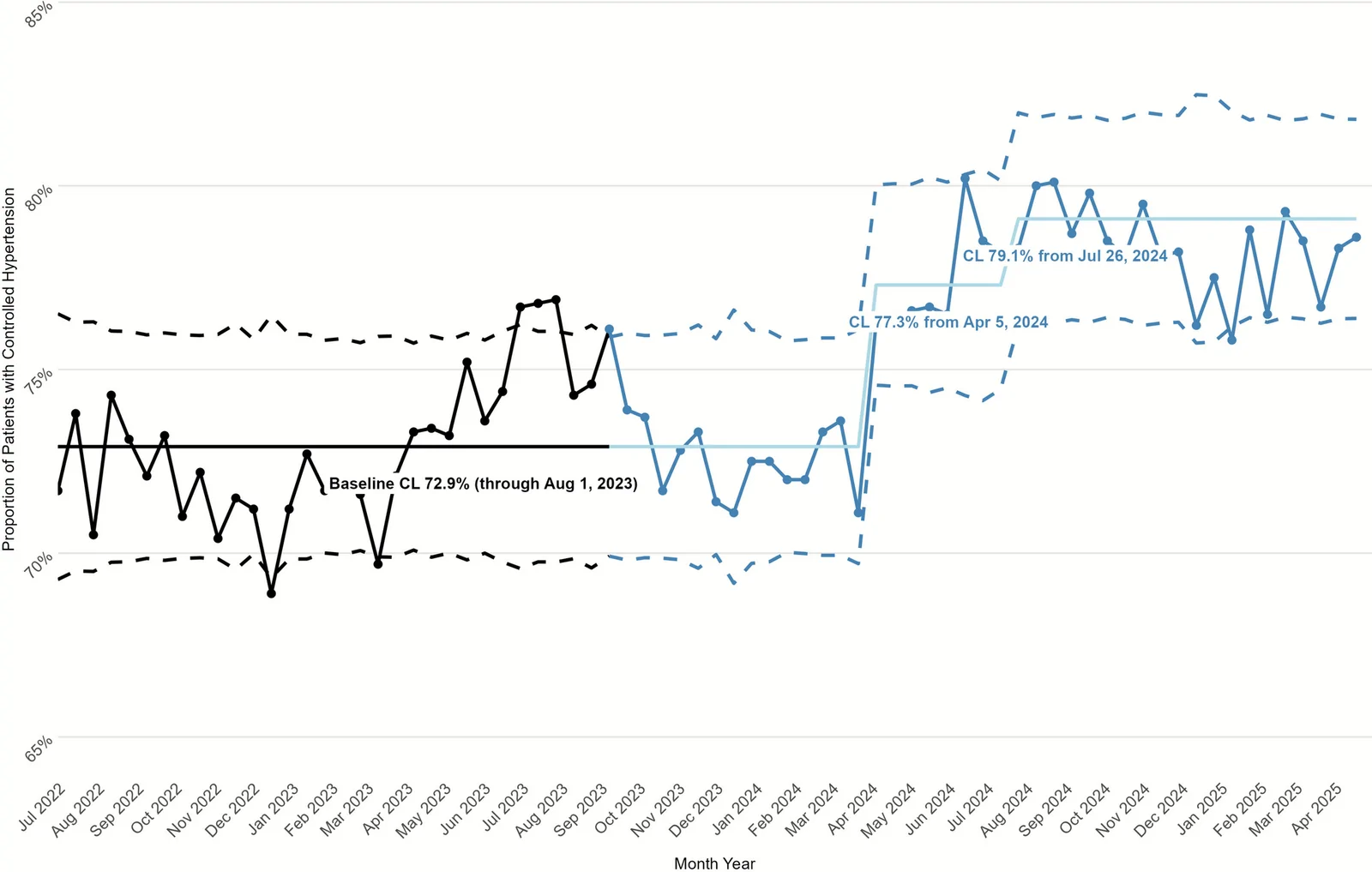
**Shewhart P-Chart of blood pressure control across all spoke sites. The Shewhart P-Chart shows the percentage of all patients across all ten spoke sites with their BP controlled during the baseline period to 18 months post-implementation. The rate of BP control is measured by the percentage of patients with systolic blood pressure < 140 mmHg and diastolic blood pressure < 90 mmHg. The y-axis indicates the rate of BP control (%), and the x-axis indicates the month and year data was collected and reported. The baseline data is black. The blue data represents the rate of BP control month to month. The upper and lower control limits are indicated by the dashed lines above and below the mean centerline.**

**Figure 3:**
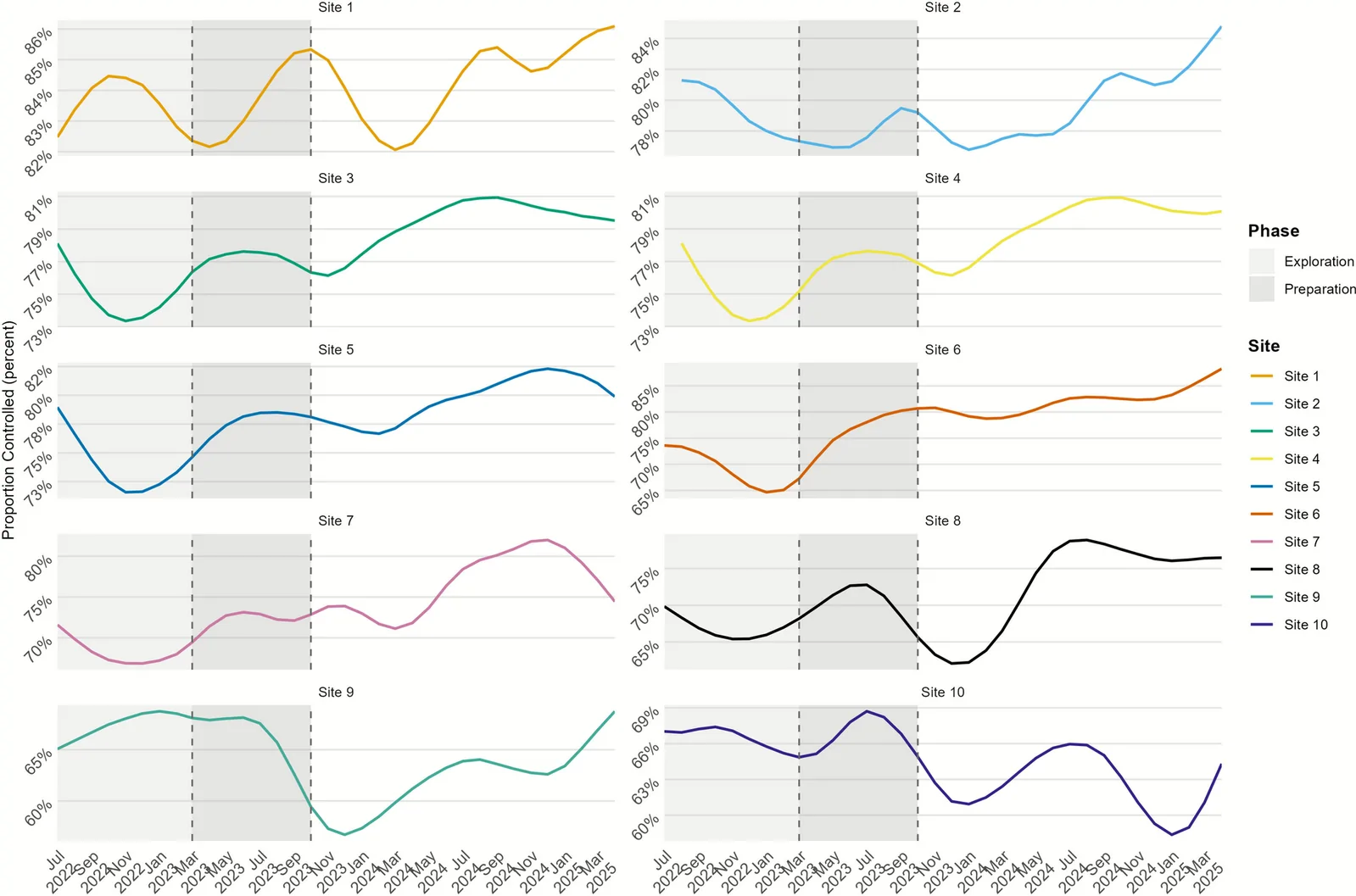
**Change in blood pressure control over time by site. The BP control rate by site during the baseline period and 18 months post-implementation. The rate of BP control is measured by the percentage of patients with systolic blood pressure < 140 mmHg and diastolic blood pressure < 90 mmHg. The y-axis indicates the proportion of patients with controlled BP (%), and the x-axis indicates the month and year data were collected and reported. The EPIS framework phase in which this data was reported is indicated by dashed gray lines, and there was some overlap between phases.**

Aggregate percentage of encounters with controlled BP is depicted in Fig. 2. The pre-implementation centerline mean was 72.9%. Two statistically significant upward shifts in the process mean were detected after ≥ 8 consecutive monthly points fell above the centerline (*p* = 0.008). The first shift occurred in April 2024, establishing a new centerline at 77.3%, followed by a second shift in July 2024 to 79.1%. The final centerline represented a 6.2% absolute and 8.5% relative improvement from baseline. Shifts exceeded expected common-cause variation, with control limits reflecting seasonally adjusted ranges. Individual site performance varied, with changes in encounter-level control rates ranging from −3.9% to + 14.6% (Fig. 3, Table 3) with several sites successfully transitioned into sustainment. Improvements corresponded to approximately 4542 additional encounters with controlled BP compared to baseline expectations.

**Table 3:** Blood Pressure Control, By Site, Baseline To 18 Months. This Table Shows The Mean Centerlines Of Blood Pressure Control BY Site And Period. For Each Site And Period, Values Are The Monthly Mean Centerline Of The Encounter-level Proportion With BP Controlled, Including All Race/ethnicity Groups (Non-hispanic White, Non-hispanic Black, Hispanic, Other, Unknown). Listed PDSA Cycles Are Not Exhaustive

Site	Baseline blood pressure control rate (%)	18-month blood pressure control rate (%)	Change in blood pressure control rate from baseline to 18 months (relative% [absolute%])	Plan-Do-Study-Act (PDSA) cycles implemented
1	83.3	84.5	1.2 [1.4]	Blood pressure re-check process created Nurse follow-up visits
2	77.7	82.7	5.0 [6.4]	Blood pressure re-check process created
3	76.9	84.1	7.9 [9.4]	Adapt patient room set up for more accurate measurement of BP
4	75.9	81.5	5.6 [7.4]	Nurse follow-up visits
5	75.3	80.6	5.3 [7.0]	Blood pressure measurement last in rooming sequence Nurse follow-up visits
6	71.9	86.5	14.6 [20.3]	Blood pressure re-check process created Nurse follow-up visits
7	69.6	81.2	11.6 [16.7]	Blood pressure re-check process created
8	69.0	76.7	7.7 [11.2]	Blood pressure measurement last in rooming sequence
9	67.7	63.8	−3.9 [−5.8]	Blood pressure re-check process created
10	66.4	66.4	0 [0]	–

### Comprehensive Patient Care

Although PDSAs for outcome consistency were not the primary emphasis, a site-level sensitivity analysis assessed BP control differences by race (Table 4). Baseline variations were evident across sites. After 18 months, six sites showed narrowing and four showed widening of the Black–White control gap. Notably, Site 9 narrowed because White control decreased more than Black control. None of the site-level sensitivity models identified a statistically significant interaction.

**Table 4:** **Blood Pressure Control Across Demographic Groups, By Site, Baseline To 18 Months. This Table Shows The Mean Percentage Of Encounters Where Blood Pressure Was Controlled At Baseline And 18-Month Post-implementation. For Each Site And Period, Values Are The Monthly Mean Centerline for Non-Hispanic White and Non-Hispanic Black Encounters; Other Groups Are Not Displayed Here. The Overall Row Is Descriptive; Inferential Conclusions Are Based On Site-level Logistic Regressions (Race × Period), In Which No Site Showed A Statistically Significant Interaction**

Site ID	Mean Non-Hispanic White baseline controlled BP (%)	Mean Non-Hispanic Black baseline controlled BP (%)	Relative Non-Hispanic Black vs Non-Hispanic White baseline gap (%)	Mean Non-Hispanic White 18-month controlled BP (%)	Mean Non-Hispanic Black 18-month controlled BP (%)	Relative Non-Hispanic Black vs Non-Hispanic White 18-month gap (%)	Difference in gap, 18-month to baseline
1	83.7	81.2	3.0	84.5	82.8	2.0	−1.0
2	78.9	73.5	6.8	80.3	76.4	4.9	−1.9
3	77.5	72.7	6.2	84.1	80.5	4.3	−1.9
4	75.5	72.6	3.8	78.9	76.8	2.7	−1.1
5	76.2	70.7	7.2	80.4	74.9	6.8	−0.4
6	72.7	69.2	4.8	83.0	75.4	9.2	+ 4.4
7	71.1	65.2	8.3	77.8	71.1	8.6	+ 0.3
8	70.2	67.0	4.6	73.2	69.2	5.5	+ 0.9
9	73.7	65.6	11.0	65.2	60.7	6.9	−4.1
10	68.9	62.8	8.9	66.8	55.0	17.7	+ 8.8
Overall	75.1	67.1	10.7	78.7	67.8	13.9	+ 3.2

### Implementation Insights

Qualitative findings related to site engagement, data use, workflow barriers, and adaptive Hub support are summarized in Table 5.

**Table 5:** **Key Findings, Outputs, And Learnings Applied EPIS Phase. The Table Below Details Key Findings Or Learnings Using The Exploration, Preparation, Implementation, A Sustainment Phases Of The EPIS Framework**

	**Hypertension-specific lessons**	**QI Hub model lessons**
**Exploration**		
	• Previous hypertension initiatives generated knowledge but lacked mechanisms for institutionalization and therefore integration must be designed “in” from the start • COVID-19-related service disruptions dissolved many previous highly successful QI initiatives	• Primary-care QI activities were historically fragmented between Family Medicine and General Internal Medicine, with limited central coordination or data infrastructure • QI work was often perceived as punitive or top-down making a collaborative, learning-oriented culture a must • Clinicians reported restricted data access and insufficient analytic capacity, signalling the necessity of a shared informatics backbone
**Preparation**		
	• Explicitly distinguishing QI from research helped position hypertension work as core clinical activity rather than optional scholarship • Self-reported site performance frequently diverged from objective data • Several sites believed they were conducting QI yet lacked structured methodology	• A relationship-centered, supportive facilitation style (vs. punitive) accelerated trust and engagement • Existing interpersonal networks were pivotal to secure early buy-in • Unlocking timely EHR data required sustained informatics negotiation and a dedicated data scientist • Formal project management capacity proved essential to coordinate the program’s complexity • Non-judgemental, in-person observation (“boots-on-the-ground”) was critical for contextual understanding and building trust
**Implementation**		
	• Framing interventions as support for extant site priorities, rather than external mandates, increased receptivity • Near real-time dashboards functioned as both a credibility lever and a learning tool, fostering progressive data literacy and perceived value • Presence of a designated site champion was indispensable for momentum and sustainability • Repetitive focus on a single clinical target risked QI fatigue amid competing organizational metrics	• Data products must be co-designed with end-users; unmet information needs undermine adoption • Continuous analytic support is required as measure specifications evolve • Incentive structures need to resonate across professional groups (e.g., medical assistants as well as physicians) • Iterative course-correction is obligatory; transparent communication about operational realities prevents misalignment • The Hub’s neutral, non-hierarchical status facilitated candid disclosure of barriers
**Sustainment**		
	• Embedding standard workflows early at both site and system levels is essential for maintenance of improvement gains • Behavioral design principle: make correct processes easier than incorrect ones	• A central repository of succinct, high-quality QI resources is necessary to refresh methodological skills • As institutional scale increases, contesting priorities multiply; ongoing alignment with executive agendas is vital for Hub longevity

## DISCUSSION

This report describes the implementation of a Regional QI Hub using a hub-and-spoke model within a statewide LHS initiative, piloting hypertension management across ten primary care sites. Over 18 months, aggregate hypertension control improved, with two statistically significant upward shifts in the SPC process mean, suggesting that networked QI support may be associated with improved chronic disease outcomes, though results varied by site.

Robust data infrastructure and tailored QI coaching, core LHS components,^7^ were cited by engaged sites as essential for identifying priorities and monitoring progress. Near real-time feedback via a centralized dashboard enabled sites to adapt interventions locally.^2^ However, operational instability, particularly staffing shortages, limited sustained engagement at several clinics, especially those serving under-resourced populations.^24–26^ These barriers echo findings from Fiscella et al., who observed that high staff turnover in Federally Qualified Health Centers impeded intervention continuity.^27^ While the Hub’s flexible support and advocacy may have helped some sites, the observational nature of this study precludes causal inference.

Our findings add to the growing literature supporting multi-component QI interventions in primary care.^27^ Similar improvements in BP control have been demonstrated in trials combining physician training, medication subsidies, and nurse-led care,^28^ or technology-driven models integrating education and standardized care protocols.^29^ While our Hub emphasized capacity building and data-enabled PDSAs rather than direct care delivery, the unifying theme across effective models is multilevel support targeting multiple system levers. Team-based care and learning collaboratives are especially valuable for sustaining improvements in complex conditions like hypertension.^30–33^

Wave 1 insights point to several future priorities: ensuring early leadership buy-in, assessing site readiness, embedding QI into daily workflows, and maintaining access to actionable data.^34–36^ For QI to be sustained, activities must integrate into routine clinical practice and be supported by time-protective structures.^35,37,38^ As the initiative expands, the Hub model must continue to adapt to local context while fostering system-wide learning. While some LHS models focus on either centralized learning or local autonomy, our experience suggests hub-and-spoke structures can bridge these by combining standard tools with site-specific flexibility. Our findings should be interpreted with caution. The 18-month evaluation period, focus on a single academic health system, and observational design limit generalizability and preclude causal conclusions. Additionally, the initiative’s multi-component nature makes it difficult to isolate the contribution of individual elements. It is also important to note that the spoke sites selected may not be representative of all primary care practices. Finally, PDSA cycles are ongoing, and continued adaptations by sites may further influence outcomes.

## CONCLUSION

In its first 18 months, OSU’s Regional QI Hub, part of a statewide LHS initiative, was associated with sustained improvements in hypertension control. Contributing factors included centralized data infrastructure, flexible QI support, and participation in a statewide learning network. Persistent staffing challenges at some sites highlighted the need for adaptive implementation strategies. Our early findings suggest that a hub-and-spoke QI model can build capacity and foster continuous learning in primary care, with further work needed to ensure sustainability, reduce differences in outcome variations, and extend the model to additional settings and conditions.

## Supplementary Information

Below is the link to the electronic supplementary material.

ESM 1 (160 KB PNG)

## Data Availability

The data supporting the findings of this study are not publicly available because they are subject to institutional restrictions.
